# Different lymph node dissection ranges during radical prostatectomy for patients with prostate cancer: a systematic review and network meta-analysis

**DOI:** 10.1186/s12957-023-02932-y

**Published:** 2023-03-06

**Authors:** Xianlu Zhang, Gejun Zhang, Jianfeng Wang, Jianbin Bi

**Affiliations:** Department of Urology Surgery, The First Hospital of China Medical University, Shenyang, 110801 China

**Keywords:** Prostate cancer, Lymph node dissection, Outcomes, Complications, Network meta-analysis

## Abstract

**Objective:**

The purpose of this network meta-analysis was to compare the effectiveness and adverse effects of limited, standard, extended, and super-extended pelvic lymph node dissection (PLND) following radical prostatectomy.

**Methods:**

This study followed the PRISMA 2020 statement. Clinical trials were searched from three electronic databases, including PubMed, the Cochrane Library, and Embase from the database’s inception to April 5, 2022. The lymph node-positive rate, biochemical recurrence-free rate, lymphocele rate, thromboembolic rate, and overall complication rate were compared by meta-analysis. Data analyses were performed using R software based on the Bayesian framework.

**Results:**

Sixteen studies involving 15,269 patients were included. All 16 studies compared the lymph node-positive rate; 5 studies compared the biochemical recurrence-free rate; 10 studies compared the lymphocele rate; 6 studies compared the thromboembolic rate, and 9 studies compared the overall complication rate. According to Bayesian analysis, the lymph node-positive rate, lymphocele rate, and overall complication rate were significantly associated with the extension of the PLND range. The limited, extended, and super-extended PLND templates showed a similar but lower biochemical recurrence-free rate and a higher thromboembolic rate than the standard template.

**Conclusions:**

The extension of the PLND range is associated with an elevated lymph node-positive rate; however, it does not improve the biochemical recurrence-free rate and correlates with an increased risk of complications, especially lymphocele. The selection of the PLND range in clinical practice should consider the oncological risk and adverse effects.

**Trial registration:**

PROSPERO (CRD42022301759).

**Supplementary Information:**

The online version contains supplementary material available at 10.1186/s12957-023-02932-y.

## Introduction

Prostate cancer (PCa) is the second most common malignancy in men. Approximately 1.4 million new cases were reported globally in 2020, with a 14.1% morbidity rate [1]. Radical prostatectomy is a common curative treatment recommended for local PCa. This open, laparoscopic, and robot-assisted approach has been widely applied and has been demonstrated beneficial to overall survival (OS) and cancer-specific survival (CSS) [2].

Pelvic lymph node dissection (PLND) is usually performed during radical prostatectomy. The European Association of Urology (EAU) guideline recommends extended PLND, which is the most accurate approach for staging pelvis-confined PCa in patients with a risk of nodal metastasis higher than 7% [2]. According to the American Urological Association (AUA) guidelines, PLND is advised for all patients with a medium-to-high risk of nodal metastasis [3]. Fossati et al. [4] classified PLND into four types: (1) limited (LPLND): obturator nodes; (2) standard (SPLND): obturator and external iliac nodes; (3) extended (EPLND): obturator, external, and internal iliac nodes; (4) super-extended (SePLND): EPLND plus common iliac, presacral, and/or other nodes.

In prostate cancer, there are still debates on the role of PLND, and its oncological benefits, potential risks and complications remain unclear. Many previous meta-analysis focus on PLND. For example, the meta-analysis by García-Perdomo et al. [5] compared the effectiveness and safety of SPLND and EPLND, and they concluded that a mild difference was evident favoring the EPLND in biochemical recurrence-free survival (HR 0.62, 95% confidence interval 0.36–0.87). Choo et al. [6] performed a meta-analysis on the same templates, and they reported a significant difference in biochemical recurrence between EPLND and SPLND (hazard ratio 0.71, 95% confidence interval 0.56–0.90). However, Fossati et al. [4] conducted a large meta-analysis of 66 included studies on all types of PLND, and they found that lymph node removal might not directly improve cancer outcomes; instead, it might result in more complications. That’s why we performed this network meta-analysis to horizontally compare the effectiveness of different PLND ranges. Besides, there is also a lack of a standard range for PLND templates, and the effectiveness and clinical benefits of different dissection ranges have not been compared.

The aim of this network meta-analysis was to evaluate the effectiveness of PLND, identify potential oncology outcomes, and elucidate post-surgery complications of various PLND ranges.

## Methods

This study was conducted following the PRISMA statement [7]. The PRISMA checklist is shown in Supplementary file 4. This study was registered on PROSPERO (Registration No. CRD42022301759, https://www.crd.york.ac.uk/PROSPERO/#recordDetails) [8].

### Search strategy

PubMed, Embase, and Cochrane Library were searched from the database inception to April 5, 2022 for studies on the effectiveness of PLND during radical prostatectomy in PCa patients. The literature search was completed by two independent reviewers, and search items mainly included ‘prostate cancer’, ‘prostatectomy’, and ‘lymph node excision’. Reference lists of retrieved articles were also searched for potential eligible studies. The detailed search strategy is provided in Supplementary file 1.

### Inclusion and exclusion criteria

Studies meeting the following criteria were included:

- Participants (P): patients were diagnosed with localized prostate cancer;
- Interventions (I): studies that applied an open, laparoscopic, or robot-assisted approach during radical prostatectomy;
- Comparisons (C): limited, extended, super-extended, or standard PLND during radical prostatectomy;
- Outcome measures (O): oncology outcomes and major complication outcomes were collected. Oncology outcomes included the lymph node-positive rate and biochemical recurrence-free rate. Major complications included lymphocele rate, thromboembolic rate, and overall complication rate;
- Types of study (S): published randomized controlled trials (RCTs) and cohort studies.

Literature review, animal study, articles in languages other than English, conference summary, repeated publication, and studies with non-RCT and non-cohort design, data incomplete or unavailable, or PLND range in the control group not based on the anatomical structure were excluded.

### Data extraction and quality assessment

Data were extracted using a pre-designed form, including first author, publication date, nation, baseline characteristics of participants (mean age, gender, body mass index, etc.), grouping, and PLND range. The risk of bias (ROB) assessment tool in the Cochrane Handbook was applied to assess the quality of included RCTs. Each RCT would be graded as ‘high risk’, ‘low risk’, or ‘unclear risk’. The quality assessment involved the following domains: random sequence generation, allocation concealment, blinding of participants and personnel, blinding of outcome assessment, incomplete outcome data, selective reporting, and other bias [9]. Cohort studies were assessed using Newcastle–Ottawa Scale (NOS). Data extraction and quality assessment were conducted by two reviewers independently. Any disagreements were resolved via discussion, a third researcher was consulted to assist in the determination if necessary.

### Statistical analysis

Data analyses were performed using the Gemtc package of R software (version 4.1.2), and Markov chain Monte Carlo (MCMC) based on the Bayesian framework was applied for modeling. Outcomes for analysis included lymph node-positive rate, biochemical recurrence-free rate, lymphocele rate, thromboembolic rate, and overall complication rate. The parameters in R were set as number of chains, 4; tuning iterations, 10,000; simulation iterations, 5000; thinning interval, 10. Odds ratio (OR) with 95% confidence interval (95%CI) was used as the effect size of dichotomous variables, and continuous data were reported as mean difference (MD) with 95% CI. A heterogeneity test was performed, and *I*^*2*^ was used to measure the heterogeneity. A random-effects model was applied when the heterogeneity among included studies was considered significant (*I*^*2*^ > 50%); otherwise (*I*^*2*^ < 50%), a fixed-effects model was adopted. Convergence of the model was evaluated by the Brooks–Gelman–Rubin method with the potential scale reduction factor (PSRF) as an evaluation indicator. PSRF values close to 1 indicated a better convergence effect of the model. The league table is listed in Supplementary file 2. The origin data collection forms, data used for all analyses, and analytic code are shown in Supplementary file 3.

## Results

### Characteristics of included studies

**Fig. 1:**
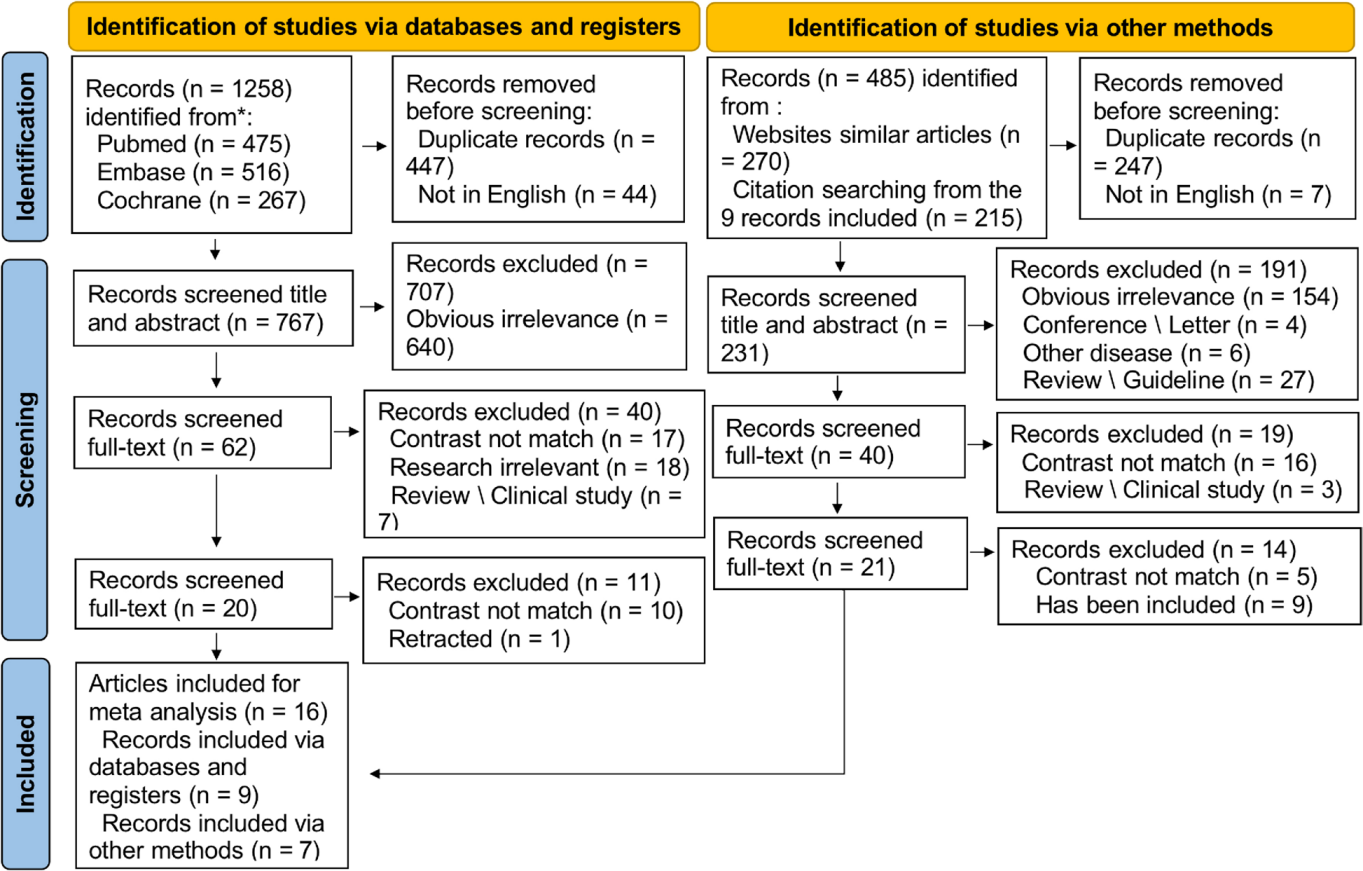
Literature selection flow chart (Sixteen studies were ultimately included in the network meta-analyses)

There were 1258 articles identified, and 9 of them met the inclusion criteria and were included. Reference lists of the nine studies were also searched, and another seven eligible studies were included. Therefore, a total of 16 studies [10–25] were included with a total of 15,269 participants. The literature selection process is shown in Fig. 1.

**Fig. 2:**
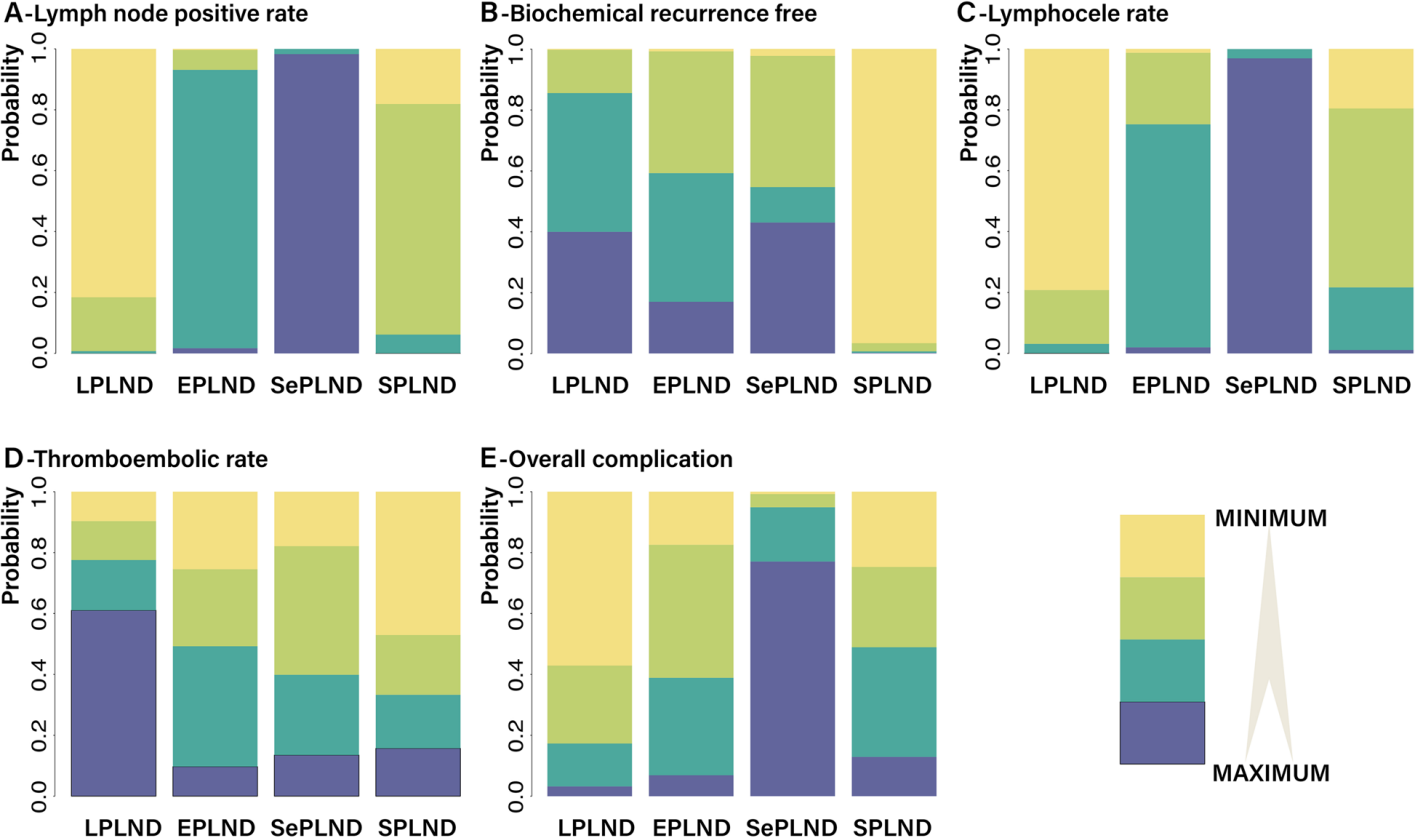
The rankogram for the effectiveness of different PLND ranges in certain outcomes

There were 2664 patients receiving limited PLND, 6141 receiving extended PLND, 1361 receiving super-extended PLND, and 5103 receiving standard PLND. The basic characteristics of the included studies were summarized in Supplementary file 2. Four nodes were compared, and the network of each outcome is shown in Supplementary file 2. The size and edge thickness of each node were weighted according to the number of participants in each comparison. The probability ranking map for outcomes is shown in Fig. 2. The PSRF value of all outcomes was 1.0, indicating fair convergence, iterative effect, and stability of the model. The results of risk of bias assessment are presented in Supplementary file 2.

### Lymph node-positive rate

Meta-analysis showed that compared with SPLND, LPLND resulted in a lower incidence of positive lymph nodes [OR = 0.72, 95%CI (0.31, 1.54)], while EPLND resulted in a higher incidence of positive lymph nodes [OR = 1.71, 95%CI (0.83, 3.33)], and SePLND had the highest incidence of positive lymph nodes [OR = 3.70, 95%CI (1.86, 7.32)].

### Biochemical recurrence-free rate

Meta-analysis revealed that compared with SPLND, all the other three PLND ranges showed lower biochemical recurrence-free rates: LPLND [OR = 0.69, 95%CI (0.50, 0.94)], EPLND [OR = 0.71, 95%CI (0.52, 0.96)], SePLND [OR = 0.70, 95%CI (0.49, 0.99)], but there were no significant differences between them.

### Lymphocele rate

Meta-analysis showed that compared with SPLND, LPLND had a lower incidence of lymphocele [OR = 0.69, 95%CI (0.29, 1.66)], EPLND had a higher incidence of lymphocele [OR = 1.29, 95%CI (0.67, 2.5)], and SePLND had the highest incidence [OR = 2.00, 95%CI (1.11, 3.70)].

### Thromboembolic rate

The results of the meta-analysis revealed that compared with SPLND, all the other three PLND ranges had a higher thromboembolic rate: LPLND (OR = 3.16; 95%CI 0.12 and 189.47), EPLND (OR = 1.42; 95%CI 0.11 and 46.61), SePLND (OR = 1.32; 95%CI 0.21 and 14.41), with no significant difference between the EPLND and SePLND.

### Overall complication rate

The results of the meta-analysis revealed that compared with SPLND, LPLND had a lower overall complication rate [OR = 0.65, 95%CI (0.09, 4.68)], while SePLND had a higher overall complication rate [OR = 2.07, 95%CI (0.04, 10.35)], and EPLND had the highest overall complication rate [OR = 0.94, 95%CI (0.16, 5.31)]. However, there were no statistically significant differences between them.

## Discussion

This is the first network meta-analysis comparing the effectiveness of different PLND ranges during prostatectomy. It was noticed that there is a limited number of RCTs among published studies. Patients receiving EPLND made up the majority of the participants (40.2%), whereas those receiving SePLND made up the least amount of participants (8.9%). The unbalanced patient distribution reflects the choice preference in clinical practice and study attraction of clinical surgeries. Furthermore, the definition of the range of PLND templates is different and often used interchangeably across the studies, such as the fuzzy boundary between ‘extended’ and ‘super-extended’. As a result, a precise definition of the PLND range is needed.

The extension of the lymph node dissection range is believed to improve the lymph node-positive rate and reduce the risk of metastases [26]. All the included studies reported the lymph node-positive rate, and their results were consistent. LPLND showed few benefits in reducing the risk of positive lymph nodes. With the extension of the PLND range, the probability rank significantly increased, which theoretically improved the prediction of prognosis. Biochemical recurrence-free rate acts as the endpoint rather than metastatic-free survival (MFS) and disease-specific survival (DSS) in the articles we included, this may related with the MFS and DSS of a large amount of patients with low- or intermediate-risk prostate cancer can be controlled at a low level, long-term follow-up could get the valid conclusion but only few articles reported relative conclusions. Five of the included studies reported the biochemical recurrence-free rate, but their results were controversial. One study [10] reported that extended lymph node dissection did not alter the biochemical outcomes. One study [11] indicated that patients with preoperative biopsy ISUP GG3-GG5 PCa (International Society of Urological Pathology grade group) who underwent EPLND showed better recurrence-free survival. Another two [12, 13] studies revealed a significant improvement in biochemical recurrence-free survival for those undergoing EPLND, and the last one [14] showed no significant difference in the biochemical recurrence rate between LPLND and EPLND. Additionally, there was no significant difference in oncological outcomes between the PLND and non-PLND groups [27]. Our network meta-analysis shows that the biochemical recurrence-free rate in the LPLND, EPLND, and SePLND groups is similar, but it is significantly higher than that in the SPLND group. To explain the different results in the SPLND group, we carefully reviewed the included studies. We found that the data on LPLND were collected from the studies by Lestingi 2020 and Touijer 2021, which are both RCTs conducted in recent years. Nevertheless, the data on SPLND were mainly extracted from a large retrospective study by Allaf 2004, in which the baseline of Gleason score 8–10 (SPLND vs EPLND 8.9% vs 23.2%) and positive surgical margin (SPLND vs EPLND 4.1% vs 10.6%) appears different from other studies. The difference in participant baseline is common in retrospective studies, and it significantly decreases the actual biochemical recurrence-free rate in the SPLND group compared with the EPLND group. Therefore, the SPLND probability in the network meta-analysis might be lower than the real probability. It can be concluded that no significant difference was observed in the biochemical recurrence-free rate between the LPLND, EPLND, and SePLND templates, and the SPLND biochemical recurrence-free probability also tended to move closer to the other three templates. Although the potential advantage of PLND in improving the biochemical recurrence-free survival has not been confirmed, it does significantly affect the lymph node-positive rate and thereby bring prognostic benefits. For those with a high risk of metastasis or confirmed metastasis, PLND might be a reliable method for prostate cancer staging, which provides useful instruction for further treatment.

On top of the oncological outcomes, PLND-associated complications are commonly reported. According to the modified Clavien classification, such complications can be classified into five grades. Grade-III or higher complications were rarely reported in the included studies. A previous study [28] found that the overall incidence of PLND-related complications was 17.4%, with lymphocele accounting for the majority of the reported cases. Other common complications included urinary anastomotic leakage, thromboembolism, and fever. There is an increasing amount of research on the complications of LPLND, SPLND, and EPLND. SPLND is reported to have a significantly higher risk of complications, which might in turn lead to prolonged hospitalization.

Lymphocele is knotty in clinical practice, and 10 of the included studies reported the incidence of lymphocele in various PLND ranges. Not all lymphoceles exhibit symptoms and require treatment. Most lymphoceles disappear spontaneously, and symptomatic lymphoceles may also disappear without any intervention. The present study shows that the incidence of lymphocele increases with the extension of the dissection range, which is in line with the findings of another study [29]. The ratio of symptomatic lymphocele to all lymphocele cases is undetermined, possibly due to the difference in the definition of lymphocele, limited ultrasound findings, and various antithrombotic medications used in different study centers. For instance, the effect of heparin prophylaxis on lymph leakage is observed in a comparative study on low molecular weight heparin preoperatively [30]. Besides, lymphocele results from the leakage of afferent lymphatic channels transected during dissection, and thus appropriate hemostatic approaches can theoretically reduce the incidence of lymphocele via sealing these channels. However, fibrin sealant agents and PK bipolar forceps are demonstrated to have no contributions to reducing lymphocele risk. In contrast, several new surgical approaches such as peritoneal flap fixation and peritoneal flap interposition can reduce the incidence of lymphocele following PLND [31, 32]. More studies are needed for the further validation of these new methods.

Thromboembolism is relatively less common but more dangerous than lymphocele. It is the second most common cause of death after cancer progression. Cancer patients accompanied by thromboembolism are associated with a 2.2-fold increase in mortality compared to those without thromboembolism. Tyritzis et al. [33] have conducted a multicenter, prospective, controlled trial, which indicates that a previous history of thrombosis, pT4 stages, and Gleason score≧ 8 are predictive factors for thromboembolic events. There is evidence that the surgery approach (open, laparoscopic, robot-assisted) and PLND ranges are associated with the risk of thromboembolism in patients undergoing prostatectomy. The possible mechanism of the association between PLND and the risk of thromboembolism is that increased bleeding leads to the formation of hematomas and lymphoceles inside a limited space, causing pressure on the iliac veins. This is a common origin of deep venous thrombosis. Moreover, excessive bleeding may also result in transfusion, which is an important factor in hypercoagulation. However, a robot-assisted technique can substantially reduce the risk of excessive bleeding and local destruction, thus lowering the thromboembolic rate. The thromboembolic rate may be indirectly impacted by the extension of PLND range, but its impact is not as significant as an open, laparoscopic, or robot-assisted surgical approach. Our network meta-analysis showed a significantly increased risk of thromboembolism in the LPLND group. To explain this outcome, we tracked back the included studies. Two studies directly compared the thromboembolic rate between LPLND and SPLND. The study by Arena et al. showed no significant difference in the thromboembolic rate, whereas the study by Yuh et al. showed a 6/204 vs. 2/202 thromboembolic rate difference between LPLND and SPLND. Therefore, the thromboembolic rate might have an indirect correlation with the PLND range and is easily affected by accident events. Further research is needed to validate the findings due to the small sample sizes.

Current studies on the PLND range indicate that more RCTs of remarkable quality are needed to explore the most appropriate range of PLND. Some studies show the extension range of PLND depends on the number of lymph nodes dissected. There is still no specific standard template and surgical procedure for different PLND ranges in clinical settings, which may lead to surgeon-related bias. Although the extension of PLND increases the diagnosis accuracy, SePLND still cannot detect all the positive lymph nodes. In addition, the risk of complications and the skill level of surgeons should be taken into account. The novelty of our study is that it is the first network meta-analysis on the PLND ranges following radical prostatectomy. Previous meta-analyses compared the PLND templates in pairs, whereas our network meta-analysis compared the four templates together to conclude comprehensive findings.

There are still some limitations to this study. Firstly, most of the included studies were cohort-designs. Secondly, the lack of subgroup analysis for clinical stages and Gleason score might lead to a bias. Thirdly, the diagnostic criteria for outcomes in different studies are also different. Lastly, we did not evaluate the publication bias because the evaluation of publication bias requires more than 10 included studies.

## Conclusion

The extension of the PLND range is associated with an increased lymph node-positive rate; however, it does not improve the biochemical recurrence-free rate and correlates with an increased risk of complications, especially lymphocele. Comprehensively considering the benefits and complications, EPLND may be the best strategy for prostatectomy, if PLND is necessary. Further clinical studies with clear PLND range templates and better methodological quality are needed to validate these findings.

## Supplementary Information

**Additional file 1: Table S1.** Search history.

**Additional file 2: Figure S1.** Network plots for included studies (Each of the 4 PLND template is represented as a node, with lines between nodes representing a comparison between 2 linked treatments). **Figure S2.** Quality assessments of the included studies. **Table S1.** Characteristics of included studies. **Table S2.** The league table of outcomes.

**Additional file 3: Tables.** The origin data collection forms and data used for all analyses. Data sheet The analytic code.

**Additional file 4. **PRISMA 2020 Checklist.

## Data Availability

The datasets used and/or analyzed during the current study are available from the corresponding author on reasonable request.

## Abbreviations

PLND: Pelvic lymph node dissection
PCa: Prostate cancer
OS: Overall survival
CSS: Cancer-specific survival
EAU: European Association of Urology
AUA: American Urological Association
ROB: Risk of bias
NOS: Newcastle–Ottawa Scale
MCMC: Markov chain Monte Carlo
OR: Odds ratio
MD: Mean difference
PSRF: Potential scale reduction factor
