# Supplementary material for: Different lymph node dissection ranges during radical prostatectomy for patients with prostate cancer: a systematic review and network meta-analysis
Source: World J Surg Oncol. 2023 Mar 6;21:80. doi: 10.1186/s12957-023-02932-y (PMC9987045; doi:10.1186/s12957-023-02932-y)
Supplement: Supplementary file 1 — Additional file 1: Table S1. Search history. [file 12957_2023_2932_MOESM1_ESM.docx]

**Additional file 1**

**Table S1** Search history

| **Pubmed** | | | | | |
| --- | --- | --- | --- | --- | --- |
| Search number | Query | | Search Details | Results | Time |
| 7 | (#4 AND #5) OR (#4 AND #6) | | (("prostatic neoplasms"[MeSH Terms] OR "prostatectomy"[MeSH Terms] OR ("Prostate Neoplasms"[Title/Abstract] OR "neoplasms prostate"[Title/Abstract] OR "neoplasm prostate"[Title/Abstract] OR "Prostate Neoplasm"[Title/Abstract] OR "neoplasms prostatic"[Title/Abstract] OR "neoplasm prostatic"[Title/Abstract] OR "Prostatic Neoplasm"[Title/Abstract] OR "Prostate Cancer"[Title/Abstract] OR "cancer prostate"[Title/Abstract] OR "cancers prostate"[Title/Abstract] OR "Prostate Cancers"[Title/Abstract] OR "Cancer of the Prostate"[Title/Abstract] OR "Prostatic Cancer"[Title/Abstract] OR "cancer prostatic"[Title/Abstract] OR "cancers prostatic"[Title/Abstract] OR "Prostatic Cancers"[Title/Abstract] OR "Cancer of Prostate"[Title/Abstract] OR "malignant prostate tumor"[Title/Abstract] OR "malignant prostate tumour"[Title/Abstract] OR "malignant prostatic tumor"[Title/Abstract] OR "malignant prostatic tumour"[Title/Abstract] OR "prostate gland cancer"[Title/Abstract] OR "prostate malignancy"[Title/Abstract] OR "prostatic malignancy"[Title/Abstract] OR "prostate neoplasia"[Title/Abstract] OR "prostate tumour"[Title/Abstract] OR "prostatic neoplasia"[Title/Abstract] OR "prostatic neoplasms"[Title/Abstract] OR "prostatic tumor"[Title/Abstract] OR "prostatic tumour"[Title/Abstract] OR ("Prostatectomies"[Title/Abstract] OR "prostatectomy suprapubic"[Title/Abstract] OR "prostatectomies suprapubic"[Title/Abstract] OR "Suprapubic Prostatectomies"[Title/Abstract] OR "Suprapubic Prostatectomy"[Title/Abstract] OR "prostatectomy retropubic"[Title/Abstract] OR "Retropubic Prostatectomies"[Title/Abstract] OR "Retropubic Prostatectomy"[Title/Abstract] OR "prostate resection"[Title/Abstract] OR "prostatic adenectomy"[Title/Abstract] OR "radical prostatectomy"[Title/Abstract] OR "total prostatectomy"[Title/Abstract]))) AND ("lymph node excision"[MeSH Terms] OR ("excision lymph node"[Title/Abstract] OR "Lymph Node Excisions"[Title/Abstract] OR "Lymphadenectomy"[Title/Abstract] OR "Lymphadenectomies"[Title/Abstract] OR "Lymph Node Dissection"[Title/Abstract] OR "dissection lymph node"[Title/Abstract] OR "dissections lymph node"[Title/Abstract] OR "Lymph Node Dissections"[Title/Abstract] OR "node dissection lymph"[Title/Abstract] OR "lymph nodal dissection"[Title/Abstract] OR "lymph node excision"[Title/Abstract] OR "lymph node extirpation"[Title/Abstract] OR "lymph node resection"[Title/Abstract] OR "lymphoadenectomy"[Title/Abstract] OR "retroperitoneal lymph node dissection"[Title/Abstract] OR "limited lymph node dissection"[Title/Abstract] OR "extended lymph node dissection"[Title/Abstract] OR "super-extended lymph node dissection"[Title/Abstract] OR "standard lymph node dissection"[Title/Abstract])) AND "random*"[Title/Abstract]) OR (("prostatic neoplasms"[MeSH Terms] OR "prostatectomy"[MeSH Terms] OR ("Prostate Neoplasms"[Title/Abstract] OR "neoplasms prostate"[Title/Abstract] OR "neoplasm prostate"[Title/Abstract] OR "Prostate Neoplasm"[Title/Abstract] OR "neoplasms prostatic"[Title/Abstract] OR "neoplasm prostatic"[Title/Abstract] OR "Prostatic Neoplasm"[Title/Abstract] OR "Prostate Cancer"[Title/Abstract] OR "cancer prostate"[Title/Abstract] OR "cancers prostate"[Title/Abstract] OR "Prostate Cancers"[Title/Abstract] OR "Cancer of the Prostate"[Title/Abstract] OR "Prostatic Cancer"[Title/Abstract] OR "cancer prostatic"[Title/Abstract] OR "cancers prostatic"[Title/Abstract] OR "Prostatic Cancers"[Title/Abstract] OR "Cancer of Prostate"[Title/Abstract] OR "malignant prostate tumor"[Title/Abstract] OR "malignant prostate tumour"[Title/Abstract] OR "malignant prostatic tumor"[Title/Abstract] OR "malignant prostatic tumour"[Title/Abstract] OR "prostate gland cancer"[Title/Abstract] OR "prostate malignancy"[Title/Abstract] OR "prostatic malignancy"[Title/Abstract] OR "prostate neoplasia"[Title/Abstract] OR "prostate tumour"[Title/Abstract] OR "prostatic neoplasia"[Title/Abstract] OR "prostatic neoplasms"[Title/Abstract] OR "prostatic tumor"[Title/Abstract] OR "prostatic tumour"[Title/Abstract] OR ("Prostatectomies"[Title/Abstract] OR "prostatectomy suprapubic"[Title/Abstract] OR "prostatectomies suprapubic"[Title/Abstract] OR "Suprapubic Prostatectomies"[Title/Abstract] OR "Suprapubic Prostatectomy"[Title/Abstract] OR "prostatectomy retropubic"[Title/Abstract] OR "Retropubic Prostatectomies"[Title/Abstract] OR "Retropubic Prostatectomy"[Title/Abstract] OR "prostate resection"[Title/Abstract] OR "prostatic adenectomy"[Title/Abstract] OR "radical prostatectomy"[Title/Abstract] OR "total prostatectomy"[Title/Abstract]))) AND ("lymph node excision"[MeSH Terms] OR ("excision lymph node"[Title/Abstract] OR "Lymph Node Excisions"[Title/Abstract] OR "Lymphadenectomy"[Title/Abstract] OR "Lymphadenectomies"[Title/Abstract] OR "Lymph Node Dissection"[Title/Abstract] OR "dissection lymph node"[Title/Abstract] OR "dissections lymph node"[Title/Abstract] OR "Lymph Node Dissections"[Title/Abstract] OR "node dissection lymph"[Title/Abstract] OR "lymph nodal dissection"[Title/Abstract] OR "lymph node excision"[Title/Abstract] OR "lymph node extirpation"[Title/Abstract] OR "lymph node resection"[Title/Abstract] OR "lymphoadenectomy"[Title/Abstract] OR "retroperitoneal lymph node dissection"[Title/Abstract] OR "limited lymph node dissection"[Title/Abstract] OR "extended lymph node dissection"[Title/Abstract] OR "super-extended lymph node dissection"[Title/Abstract] OR "standard lymph node dissection"[Title/Abstract])) AND "cohort"[Title/Abstract]) | 475 | 0:18:14 |
| 6 | cohort[Title/Abstract] | | "cohort"[Title/Abstract] | 678,587 | 0:17:35 |
| 5 | random*[Title/Abstract] | | "random*"[Title/Abstract] | 1,307,866 | 0:17:11 |
| 4 | (#1 OR #2) AND #3 | | ("prostatic neoplasms"[MeSH Terms] OR "prostatectomy"[MeSH Terms] OR ("Prostate Neoplasms"[Title/Abstract] OR "neoplasms prostate"[Title/Abstract] OR "neoplasm prostate"[Title/Abstract] OR "Prostate Neoplasm"[Title/Abstract] OR "neoplasms prostatic"[Title/Abstract] OR "neoplasm prostatic"[Title/Abstract] OR "Prostatic Neoplasm"[Title/Abstract] OR "Prostate Cancer"[Title/Abstract] OR "cancer prostate"[Title/Abstract] OR "cancers prostate"[Title/Abstract] OR "Prostate Cancers"[Title/Abstract] OR "Cancer of the Prostate"[Title/Abstract] OR "Prostatic Cancer"[Title/Abstract] OR "cancer prostatic"[Title/Abstract] OR "cancers prostatic"[Title/Abstract] OR "Prostatic Cancers"[Title/Abstract] OR "Cancer of Prostate"[Title/Abstract] OR "malignant prostate tumor"[Title/Abstract] OR "malignant prostate tumour"[Title/Abstract] OR "malignant prostatic tumor"[Title/Abstract] OR "malignant prostatic tumour"[Title/Abstract] OR "prostate gland cancer"[Title/Abstract] OR "prostate malignancy"[Title/Abstract] OR "prostatic malignancy"[Title/Abstract] OR "prostate neoplasia"[Title/Abstract] OR "prostate tumour"[Title/Abstract] OR "prostatic neoplasia"[Title/Abstract] OR "prostatic neoplasms"[Title/Abstract] OR "prostatic tumor"[Title/Abstract] OR "prostatic tumour"[Title/Abstract] OR ("Prostatectomies"[Title/Abstract] OR "prostatectomy suprapubic"[Title/Abstract] OR "prostatectomies suprapubic"[Title/Abstract] OR "Suprapubic Prostatectomies"[Title/Abstract] OR "Suprapubic Prostatectomy"[Title/Abstract] OR "prostatectomy retropubic"[Title/Abstract] OR "Retropubic Prostatectomies"[Title/Abstract] OR "Retropubic Prostatectomy"[Title/Abstract] OR "prostate resection"[Title/Abstract] OR "prostatic adenectomy"[Title/Abstract] OR "radical prostatectomy"[Title/Abstract] OR "total prostatectomy"[Title/Abstract]))) AND ("lymph node excision"[MeSH Terms] OR ("excision lymph node"[Title/Abstract] OR "Lymph Node Excisions"[Title/Abstract] OR "Lymphadenectomy"[Title/Abstract] OR "Lymphadenectomies"[Title/Abstract] OR "Lymph Node Dissection"[Title/Abstract] OR "dissection lymph node"[Title/Abstract] OR "dissections lymph node"[Title/Abstract] OR "Lymph Node Dissections"[Title/Abstract] OR "node dissection lymph"[Title/Abstract] OR "lymph nodal dissection"[Title/Abstract] OR "lymph node excision"[Title/Abstract] OR "lymph node extirpation"[Title/Abstract] OR "lymph node resection"[Title/Abstract] OR "lymphoadenectomy"[Title/Abstract] OR "retroperitoneal lymph node dissection"[Title/Abstract] OR "limited lymph node dissection"[Title/Abstract] OR "extended lymph node dissection"[Title/Abstract] OR "super-extended lymph node dissection"[Title/Abstract] OR "standard lymph node dissection"[Title/Abstract])) | 3,439 | 0:16:49 |
| 3 | (Lymph Node Excision[MeSH Terms]) OR ("Excision, Lymph Node"[Title/Abstract] OR "Excisions, Lymph Node"[Title/Abstract] OR "Lymph Node Excisions"[Title/Abstract] OR "Lymphadenectomy"[Title/Abstract] OR "Lymphadenectomies"[Title/Abstract] OR "Lymph Node Dissection"[Title/Abstract] OR "Dissection, Lymph Node"[Title/Abstract] OR "Dissections, Lymph Node"[Title/Abstract] OR "Lymph Node Dissections"[Title/Abstract] OR "Node Dissection, Lymph"[Title/Abstract] OR "Node Dissections, Lymph"[Title/Abstract] OR "lymph nodal dissection"[Title/Abstract] OR "lymph node dissection, retroperitoneal"[Title/Abstract] OR "lymph node excision"[Title/Abstract] OR "lymph node extirpation"[Title/Abstract] OR "lymph node resection"[Title/Abstract] OR "lymphoadenectomy"[Title/Abstract] OR "retroperitoneal lymph node dissection"[Title/Abstract] OR "limited lymph node dissection"[Title/Abstract] OR "extended lymph node dissection"[Title/Abstract] OR "super-extended lymph node dissection"[Title/Abstract] OR "expanded lymph node dissection"[Title/Abstract] OR "standard lymph node dissection"[Title/Abstract]) | | "lymph node excision"[MeSH Terms] OR "excision lymph node"[Title/Abstract] OR "Lymph Node Excisions"[Title/Abstract] OR "Lymphadenectomy"[Title/Abstract] OR "Lymphadenectomies"[Title/Abstract] OR "Lymph Node Dissection"[Title/Abstract] OR "dissection lymph node"[Title/Abstract] OR "dissections lymph node"[Title/Abstract] OR "Lymph Node Dissections"[Title/Abstract] OR "node dissection lymph"[Title/Abstract] OR "lymph nodal dissection"[Title/Abstract] OR "lymph node excision"[Title/Abstract] OR "lymph node extirpation"[Title/Abstract] OR "lymph node resection"[Title/Abstract] OR "lymphoadenectomy"[Title/Abstract] OR "retroperitoneal lymph node dissection"[Title/Abstract] OR "limited lymph node dissection"[Title/Abstract] OR "extended lymph node dissection"[Title/Abstract] OR "super-extended lymph node dissection"[Title/Abstract] OR "standard lymph node dissection"[Title/Abstract] | 70,174 | 0:16:29 |
| 2 | ("Prostate Neoplasms"[Title/Abstract] OR "Neoplasms, Prostate"[Title/Abstract] OR "Neoplasm, Prostate"[Title/Abstract] OR "Prostate Neoplasm"[Title/Abstract] OR "Neoplasms, Prostatic"[Title/Abstract] OR "Neoplasm, Prostatic"[Title/Abstract] OR "Prostatic Neoplasm"[Title/Abstract] OR "Prostate Cancer"[Title/Abstract] OR "Cancer, Prostate"[Title/Abstract] OR "Cancers, Prostate"[Title/Abstract] OR "Prostate Cancers"[Title/Abstract] OR "Cancer of the Prostate"[Title/Abstract] OR "Prostatic Cancer"[Title/Abstract] OR "Cancer, Prostatic"[Title/Abstract] OR "Cancers, Prostatic"[Title/Abstract] OR "Prostatic Cancers"[Title/Abstract] OR "Cancer of Prostate"[Title/Abstract] OR "malignant prostate tumor"[Title/Abstract] OR "malignant prostate tumour"[Title/Abstract] OR "malignant prostatic tumor"[Title/Abstract] OR "malignant prostatic tumour"[Title/Abstract] OR "prostate gland cancer"[Title/Abstract] OR "prostate malignancy"[Title/Abstract] OR "prostate malignant neoplasm"[Title/Abstract] OR "prostate malignant tumor"[Title/Abstract] OR "prostate malignant tumour"[Title/Abstract] OR "prostatic malignancy"[Title/Abstract] OR "prostate gland tumor"[Title/Abstract] OR "prostate gland tumour"[Title/Abstract] OR "prostate neoplasia"[Title/Abstract] OR "prostate tumour"[Title/Abstract] OR "prostatic neoplasia"[Title/Abstract] OR "prostatic neoplasms"[Title/Abstract] OR "prostatic tumor"[Title/Abstract] OR "prostatic tumour"[Title/Abstract]) OR ("Prostatectomies"[Title/Abstract] OR "Prostatectomy, Suprapubic"[Title/Abstract] OR "Prostatectomies, Suprapubic"[Title/Abstract] OR "Suprapubic Prostatectomies"[Title/Abstract] OR "Suprapubic Prostatectomy"[Title/Abstract] OR "Prostatectomy, Retropubic"[Title/Abstract] OR "Prostatectomies, Retropubic"[Title/Abstract] OR "Retropubic Prostatectomies"[Title/Abstract] OR "Retropubic Prostatectomy"[Title/Abstract] OR "prostate adenectomy"[Title/Abstract] OR "prostate resection"[Title/Abstract] OR "prostatic adenectomy"[Title/Abstract] OR "radical prostatectomy"[Title/Abstract] OR "total prostatectomy"[Title/Abstract] | | "Prostate Neoplasms"[Title/Abstract] OR "neoplasms prostate"[Title/Abstract] OR "neoplasm prostate"[Title/Abstract] OR "Prostate Neoplasm"[Title/Abstract] OR "neoplasms prostatic"[Title/Abstract] OR "neoplasm prostatic"[Title/Abstract] OR "Prostatic Neoplasm"[Title/Abstract] OR "Prostate Cancer"[Title/Abstract] OR "cancer prostate"[Title/Abstract] OR "cancers prostate"[Title/Abstract] OR "Prostate Cancers"[Title/Abstract] OR "Cancer of the Prostate"[Title/Abstract] OR "Prostatic Cancer"[Title/Abstract] OR "cancer prostatic"[Title/Abstract] OR "cancers prostatic"[Title/Abstract] OR "Prostatic Cancers"[Title/Abstract] OR "Cancer of Prostate"[Title/Abstract] OR "malignant prostate tumor"[Title/Abstract] OR "malignant prostate tumour"[Title/Abstract] OR "malignant prostatic tumor"[Title/Abstract] OR "malignant prostatic tumour"[Title/Abstract] OR "prostate gland cancer"[Title/Abstract] OR "prostate malignancy"[Title/Abstract] OR "prostatic malignancy"[Title/Abstract] OR "prostate neoplasia"[Title/Abstract] OR "prostate tumour"[Title/Abstract] OR "prostatic neoplasia"[Title/Abstract] OR "prostatic neoplasms"[Title/Abstract] OR "prostatic tumor"[Title/Abstract] OR "prostatic tumour"[Title/Abstract] OR "Prostatectomies"[Title/Abstract] OR "prostatectomy suprapubic"[Title/Abstract] OR "prostatectomies suprapubic"[Title/Abstract] OR "Suprapubic Prostatectomies"[Title/Abstract] OR "Suprapubic Prostatectomy"[Title/Abstract] OR "prostatectomy retropubic"[Title/Abstract] OR "Retropubic Prostatectomies"[Title/Abstract] OR "Retropubic Prostatectomy"[Title/Abstract] OR "prostate resection"[Title/Abstract] OR "prostatic adenectomy"[Title/Abstract] OR "radical prostatectomy"[Title/Abstract] OR "total prostatectomy"[Title/Abstract] | 150,848 | 0:15:50 |
| 1 | (Prostatic Neoplasms[MeSH Terms]) OR (Prostatectomy[MeSH Terms]) | | "prostatic neoplasms"[MeSH Terms] OR "prostatectomy"[MeSH Terms] | 155,367 | 0:05:00 |
| **Embase** | | | | | |
| No. | Query | | | Result | Date |
| #15 | #13 OR #14 | | | 516 | 5-Apr-22 |
| #14 | #10 AND #12 | | | 339 | 5-Apr-22 |
| #13 | #10 AND #11 | | | 196 | 5-Apr-22 |
| #12 | 'cohort':ti,ab,kw | | | 1146446 | 5-Apr-22 |
| #11 | 'random*':ti,ab,kw | | | 1778625 | 5-Apr-22 |
| #10 | #9 AND 'article'/it | | | 3447 | 5-Apr-22 |
| #9 | #5 AND #8 | | | 7180 | 5-Apr-22 |
| #8 | #6 OR #7 | | | 101105 | 5-Apr-22 |
| #7 | 'lymph node dissection':exp | | | 90609 | 5-Apr-22 |
| #6 | 'excision, lymph node':ab,ti,kw OR 'excisions, lymph node':ab,ti,kw OR 'lymph node excisions':ab,ti,kw OR 'lymphadenectomy':ab,ti,kw OR 'lymphadenectomies':ab,ti,kw OR 'lymph node dissection':ab,ti,kw OR 'dissection, lymph node':ab,ti,kw OR 'dissections, lymph node':ab,ti,kw OR 'lymph node dissections':ab,ti,kw OR 'node dissection, lymph':ab,ti,kw OR 'node dissections, lymph':ab,ti,kw OR 'lymph nodal dissection':ab,ti,kw OR 'lymph node dissection, retroperitoneal':ab,ti,kw OR 'lymph node excision':ab,ti,kw OR 'lymph node extirpation':ab,ti,kw OR 'lymph node resection':ab,ti,kw OR 'lymphoadenectomy':ab,ti,kw OR 'retroperitoneal lymph node dissection':ab,ti,kw OR 'limited lymph node dissection':ab,ti,kw OR 'extended lymph node dissection':ab,ti,kw OR 'super-extended lymph node dissection':ab,ti,kw OR 'expanded lymph node dissection':ab,ti,kw OR 'standard lymph node dissection':ab,ti,kw | | | 60891 | 5-Apr-22 |
| #5 | #1 OR #2 OR #3 OR #4 | | | 319671 | 5-Apr-22 |
| #4 | 'prostatectomy':exp | | | 67950 | 5-Apr-22 |
| #3 | 'prostate tumor':exp | | | 276793 | 5-Apr-22 |
| #2 | 'prostate cancer':exp | | | 249456 | 5-Apr-22 |
| #1 | 'prostate neoplasms':ab,ti,kw OR 'neoplasms, prostate':ab,ti,kw OR 'neoplasm, prostate':ab,ti,kw OR 'prostate neoplasm':ab,ti,kw OR 'neoplasms, prostatic':ab,ti,kw OR 'neoplasm, prostatic':ab,ti,kw OR 'prostatic neoplasm':ab,ti,kw OR 'prostate cancer':ab,ti,kw OR 'cancer, prostate':ab,ti,kw OR 'cancers, prostate':ab,ti,kw OR 'prostate cancers':ab,ti,kw OR 'cancer of the prostate':ab,ti,kw OR 'prostatic cancer':ab,ti,kw OR 'cancer, prostatic':ab,ti,kw OR 'cancers, prostatic':ab,ti,kw OR 'prostatic cancers':ab,ti,kw OR 'cancer of prostate':ab,ti,kw OR 'malignant prostate tumor':ab,ti,kw OR 'malignant prostate tumour':ab,ti,kw OR 'malignant prostatic tumor':ab,ti,kw OR 'malignant prostatic tumour':ab,ti,kw OR 'prostate gland cancer':ab,ti,kw OR 'prostate malignancy':ab,ti,kw OR 'prostate malignant neoplasm':ab,ti,kw OR 'prostate malignant tumor':ab,ti,kw OR 'prostate malignant tumour':ab,ti,kw OR 'prostatic malignancy':ab,ti,kw OR 'prostate gland tumor':ab,ti,kw OR 'prostate gland tumour':ab,ti,kw OR 'prostate neoplasia':ab,ti,kw OR 'prostate tumour':ab,ti,kw OR 'prostatic neoplasia':ab,ti,kw OR 'prostatic neoplasms':ab,ti,kw OR 'prostatic tumor':ab,ti,kw OR 'prostatic tumour':ab,ti,kw OR 'prostatectomies':ab,ti,kw OR 'prostatectomy, suprapubic':ab,ti,kw OR 'prostatectomies, suprapubic':ab,ti,kw OR 'suprapubic prostatectomies':ab,ti,kw OR 'prostatectomy, retropubic':ab,ti,kw OR 'prostatectomies, retropubic':ab,ti,kw OR 'retropubic prostatectomies':ab,ti,kw OR 'retropubic prostatectomy':ab,ti,kw OR 'suprapubic prostatectomy':ab,ti,kw OR 'prostate adenectomy':ab,ti,kw OR 'prostate resection':ab,ti,kw OR 'prostatic adenectomy':ab,ti,kw OR 'radical prostatectomy':ab,ti,kw OR 'total prostatectomy':ab,ti,kw | | | 228243 | 5-Apr-22 |
| **Cochrane** | | | | | |
| No. | | Query | | Result | |
| #1 | | (‘Prostate Neoplasms’ OR ‘Neoplasms, Prostate’ OR ‘Neoplasm, Prostate’ OR ‘Prostate Neoplasm’ OR ‘Neoplasms, Prostatic’ OR ‘Neoplasm, Prostatic’ OR ‘Prostatic Neoplasm’ OR ‘Prostate Cancer’ OR ‘Cancer, Prostate’ OR ‘Cancers, Prostate’ OR ‘Prostate Cancers’ OR ‘Cancer of the Prostate’ OR ‘Prostatic Cancer’ OR ‘Cancer, Prostatic’ OR ‘Cancers, Prostatic’ OR ‘Prostatic Cancers’ OR ‘Cancer of Prostate’ OR ‘malignant prostate tumor’ OR ‘malignant prostate tumour’ OR ‘malignant prostatic tumor’ OR ‘malignant prostatic tumour’ OR ‘prostate gland cancer’ OR ‘prostate malignancy’ OR ‘prostate malignant neoplasm’ OR ‘prostate malignant tumor’ OR ‘prostate malignant tumour’ OR ‘prostatic malignancy’ OR ‘prostate gland tumor’ OR ‘prostate gland tumour’ OR ‘prostate neoplasia’ OR ‘prostate tumour’ OR ‘prostatic neoplasia’ OR ‘prostatic neoplasms’ OR ‘prostatic tumor’ OR ‘prostatic tumour’ OR ‘Prostatectomies’ OR ‘Prostatectomy, Suprapubic’ OR ‘Prostatectomies, Suprapubic’ OR ‘Suprapubic Prostatectomies’ OR ‘Suprapubic Prostatectomy’ OR ‘Prostatectomy, Retropubic’ OR ‘Prostatectomies, Retropubic’ OR ‘Retropubic Prostatectomies’ OR ‘Retropubic Prostatectomy’ OR ‘Retropubic Prostatectomy’ OR ‘Suprapubic Prostatectomy’ OR ‘prostate adenectomy’ OR ‘prostate resection’ OR ‘prostatic adenectomy’ OR ‘radical prostatectomy’ OR ‘total prostatectomy’):ti,ab,kw | | 18349 | |
| #2 | | MeSH descriptor: [Prostatic Neoplasms] explode all trees | | 6079 | |
| #3 | | MeSH descriptor: [Prostatectomy] explode all trees | | 1873 | |
| #4 | | #1 OR #2 OR #3 | | 18583 | |
| #5 | | (‘Excision, Lymph Node’ OR ‘Excisions, Lymph Node’ OR ‘Lymph Node Excisions’ OR ‘Lymphadenectomy’ OR ‘Lymphadenectomies’ OR ‘Lymph Node Dissection’ OR ‘Dissection, Lymph Node’ OR ‘Dissections, Lymph Node’ OR ‘Lymph Node Dissections’ OR ‘Node Dissection, Lymph’ OR ‘Node Dissections, Lymph’ OR ‘lymph nodal dissection’ OR ‘lymph node dissection, retroperitoneal’ OR ‘lymph node excision’ OR ‘lymph node extirpation’ OR ‘lymph node resection’ OR ‘lymphoadenectomy’ OR ‘retroperitoneal lymph node dissection’ OR ‘limited lymph node dissection’ OR ‘extended lymph node dissection’ OR ‘super-extended lymph node dissection’ OR ‘expanded lymph node dissection’ OR ‘standard lymph node dissection’):ti,ab,kw | | 6085 | |
| #6 | | MeSH descriptor: [Lymph Node Excision] explode all trees | | 1436 | |
| #7 | | #5 OR #6 | | 6281 | |
| #8 | | #4 AND #7 | | 351 | |
| #9 | | (random*):ti,ab,kw | | 1129902 | |
| #10 | | (cohort):ti,ab,kw | | 61015 | |
| #11 | | #8 AND #9 | | 259 | |
| #12 | | #8 AND #10 | | 36 | |
| #13 | | #11 OR #12 | | 267 | |
