# Supplementary material for: Different lymph node dissection ranges during radical prostatectomy for patients with prostate cancer: a systematic review and network meta-analysis
Source: World J Surg Oncol. 2023 Mar 6;21:80. doi: 10.1186/s12957-023-02932-y (PMC9987045; doi:10.1186/s12957-023-02932-y)
Supplement: Supplementary file 2 — Additional file 2: Figure S1. Network plots for included studies (Each of the 4 PLND template is represented as a node, with lines between nodes representing a comparison between 2 linked treatments). Figure S2. Quality assessments of the included studies. Table S1. Characteristics of included studies. Table S2. The league table of outcomes. [file 12957_2023_2932_MOESM2_ESM.docx]

**Additional file 2**


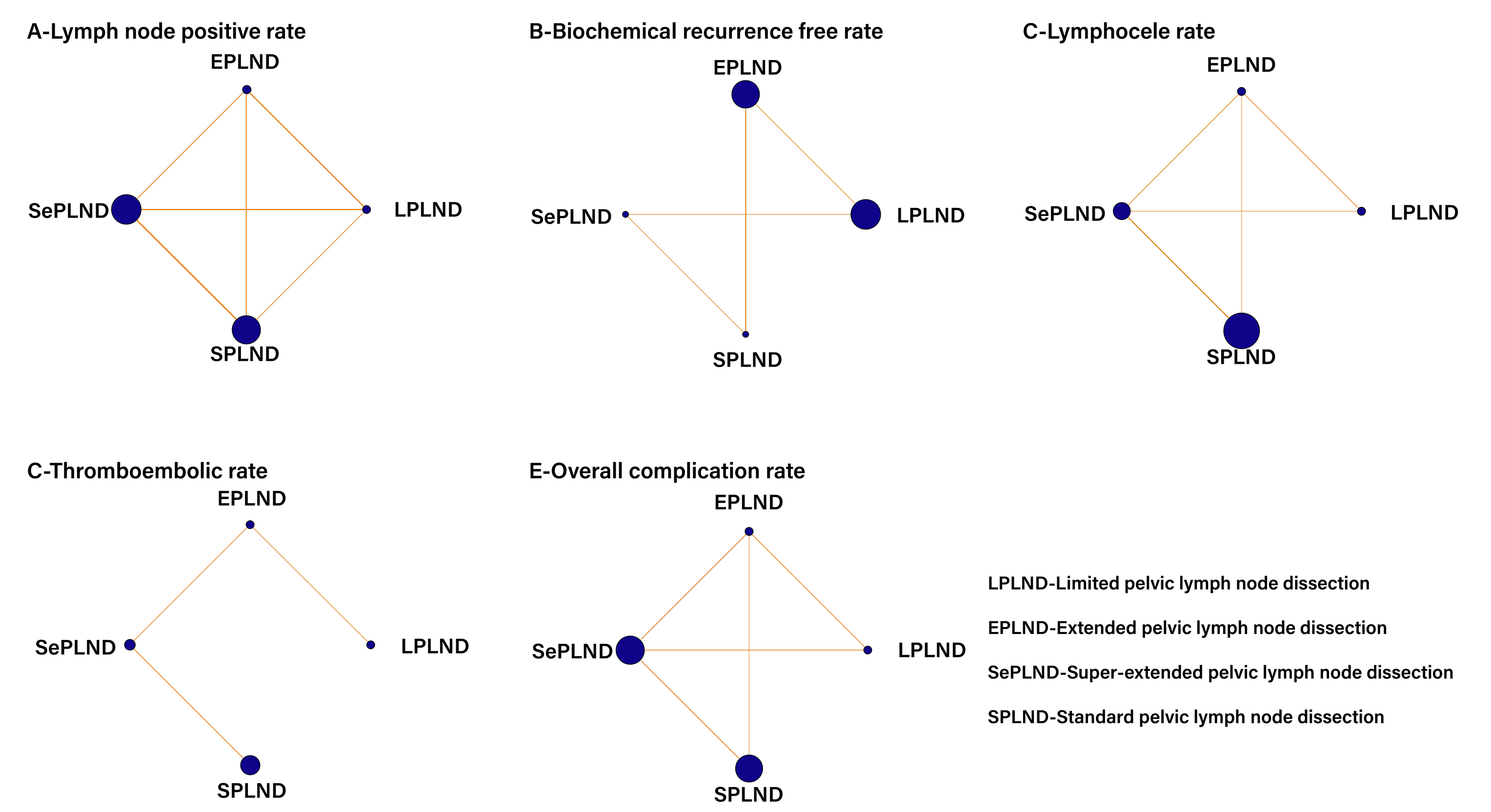


**Fig. S1** Network plots for included studies (Comparison networks were explored by representing each of the 4 PLND template as a node, with lines between nodes representing a comparison between 2 linked treatments)


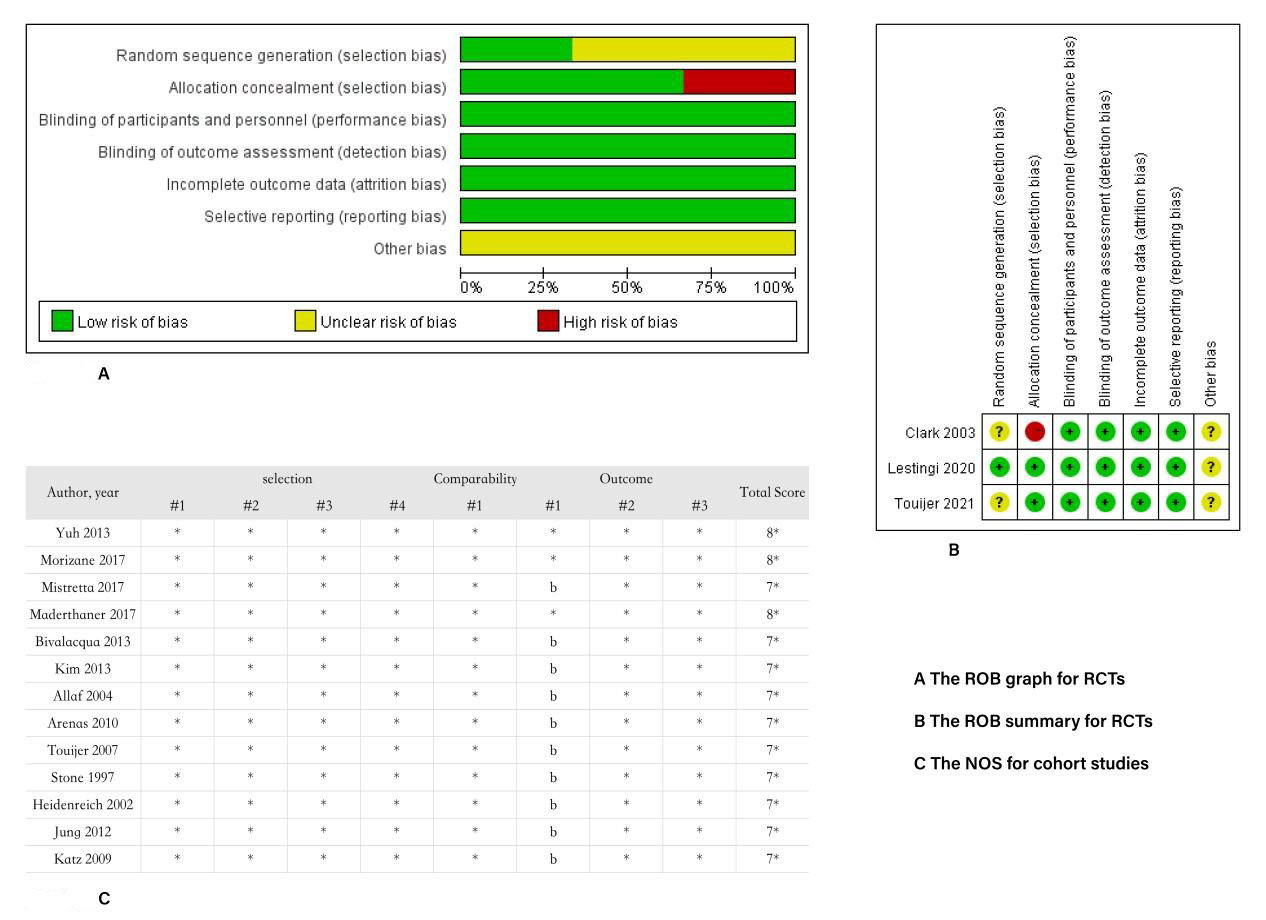


**Fig. S2** Quality assessments of the included studies

**Table S1** Characteristics of included studies

| Study | Year | Country | Patients | Sample size of each arm | | | | Outcomes |
| --- | --- | --- | --- | --- | --- | --- | --- | --- |
|  |  |  |  | LPLND  (n = 2664) | EPLND  (n = 6141) | SePLND  (n = 1361) | SPLND  (n = 5103) |  |
| Touijer et al.[12] | 2021 | USA | localized PCa | 700 | 740 | NA | NA | A,B,E |
| Lestingi et al.[9] | 2021 | USA | Intermediate- or high-risk PCa | 150 | NA | 150 | NA | A,B |
| Clark et al.[13] | 2003 | USA | PCa | NA | NA | 123 | 123 | A,C,D |
| Yuh et al.[14] | 2013 | USA | Intermediate- or high-risk PCa | 204 | 202 | NA | NA | A,C,D,E |
| Morizane et al.[15] | 2018 | Japan | PCa | 902 | NA | 431 | NA | A,C,E |
| Mistretta et al.[16] | 2017 | Italy | localized PCa | NA | 75 | NA | 109 | A,C,E |
| Maderthaner et al.[17] | 2018 | Switzerland | localized PCa | NA | 485 | 268 | NA | A,C,D,E |
| Bivalacqua et al.[10] | 2013 | USA | PCa with >1 positive LNs | NA | 2279 | NA | 1986 | A,B |
| Kim et al.[8] | 2013 | South Korea | Intermediate- or high-risk localized PCa | NA | NA | 170 | 294 | A,B,C,E |
| Allaf et al.[11] | 2004 | USA | localized PCa | NA | 2135 | NA | 1865 | A,B |
| Arenas et al.[18] | 2010 | Germany | localized PCa | 381 | 163 | NA | NA | A,C,D |
| Touijer et al.[19] | 2007 | USA | localized (cT1–cT3a) PCa | 177 | NA | NA | 471 | A |
| Stone et al.[20] | 1997 | USA | T1-T3 PCa | 150 | NA | 39 | NA | A,E |
| Heidenreich et al.[21] | 2002 | Germany | localized PCa | NA | NA | 103 | 100 | A,C,D,E |
| Jung et al.[22] | 2012 | South Korea | PCa | NA | NA | 45 | 155 | A,C |
| Katz et al.[23] | 2010 | USA | localized PCa | NA | 62 | 32 | NA | A,C,D,E |

A-Lymph node positive rate; B-Biochemical recurrence free rate; C-Lymphocele rate; D-Thromboembolism rate; E-Overall complication rate.

**Table S2** The league table of outcomes

| Positive lymph node rate | | | |
| --- | --- | --- | --- |
| LPLND |  |  |  |
| 0.42 (0.21, 0.83) | EPLND |  |  |
| 0.19 (0.09, 0.39) | 0.46 (0.22, 0.94) | SePLND |  |
| 0.72 (0.31, 1.54) | 1.71 (0.83, 3.33) | 3.70 (1.86, 7.32) | SPLND |
| Biochemical recurrence free rate | | | |
| LPLND |  |  |  |
| 0.97 (0.87, 1.08) | EPLND |  |  |
| 0.98 (0.72, 1.35) | 1.01 (0.73, 1.39) | SePLND |  |
| 0.69 (0.50, 0.94) | 0.71 (0.52, 0.96) | 0.70 (0.49, 0.99) | SPLND |
| Lymphocele rate | | | |
| LPLND |  |  |  |
| 0.53 (0.26, 1.05) | EPLND |  |  |
| 0.35 (0.17, 0.67) | 0.64 (0.42, 0.98) | SePLND |  |
| 0.69 (0.29, 1.66) | 1.29 (0.67, 2.50) | 2.00 (1.11, 3.70) | SPLND |
| Thromboembolism rate | | | |
| LPLND |  |  |  |
| 2.21 (0.28, 19.37) | EPLND |  |  |
| 2.39 (0.15, 59.11) | 1.06 (0.17, 10.94) | SePLND |  |
| 3.16 (0.12, 189.47) | 1.42 (0.11, 46.61) | 1.32 (0.21, 14.14) | SPLND |
| Overall complication rate | | | |
| LPLND |  |  |  |
| 0.70 (0.15, 2.97) | EPLND |  |  |
| 0.32 (0.07, 1.34) | 0.45 (0.11, 1.76) | SePLND |  |
| 0.65 (0.09, 4.68) | 0.94 (0.16, 5.31) | 2.07 (0.44, 10.35) | SPLND |
| Numbers in the table are the RR value of different comparisons of interventions. | | | |
