# Supplementary material for: Different lymph node dissection ranges during radical prostatectomy for patients with prostate cancer: a systematic review and network meta-analysis
Source: World J Surg Oncol. 2023 Mar 6;21:80. doi: 10.1186/s12957-023-02932-y (PMC9987045; doi:10.1186/s12957-023-02932-y)
Supplement: Supplementary file 3 — Additional file 3: Tables. The origin data collection forms and data used for all analyses. Data sheet The analytic code. [file 12957_2023_2932_MOESM3_ESM.docx]

**Additional file 3**

**The origin data collection forms and data used for all analyses:**

| Studies regarding Lymph node positive rate | Sample size of each arm | | | |
| --- | --- | --- | --- | --- |
|  | LPLND  (n = 211) | EPLND  (n = 396) | SePLND  (n =227) | SPLND  (n = 143) |
| Touijer et al. | 700(84) | 740(104) | NA | NA |
| Lestingi et al. | 150(5) | NA | 150(25) | NA |
| Clark et al. | NA | NA | 123(4) | 123(3) |
| Yuh et al. | 204(24) | 202(8) | NA | NA |
| Morizane et al. | 902(5) | NA | 431(53) | NA |
| Mistretta et al. | NA | 75(22) | NA | 109(14) |
| Maderthaner et al. | NA | 485(80) | 268(72) | NA |
| Bivalacqua et al. | NA | 2279(73) | NA | 1986(21) |
| Kim et al. | NA | NA | 170(23) | 294(10) |
| Allaf et al. | NA | 2135(68) | NA | 1865(21) |
| Arenas et al. | 381(72) | 163(40) | NA | NA |
| Touijer et al. | 177(7) | NA | NA | 471(54) |
| Stone et al. | 150(14) | NA | 39(9) | NA |
| Heidenreich et al. | NA | NA | 103(27) | 100(12) |
| Jung et al. | NA | NA | 45(10) | 155(8) |
| Katz et al. | NA | 62(1) | 32(4) | NA |

The number indicate the total patients and the number in brackets are patients with positive outcome.

| Studies regarding Biochemical recurrence-free rate | PLND Type | Diff | Std.err |
| --- | --- | --- | --- |
| Touijer et al. | EPLND | 0.039221 | 0.054166 |
|  | LPLND |  |  |
| Lestingi et al. | SePLND | -0.09431 | 0.188691 |
|  | LPLND |  |  |
| Bivalacqua et al. | EPLND | -0.56387 | 0.304844 |
|  | SPLND |  |  |
| Kim et al. | SeOLND | -0.16252 | 0.245258 |
|  | SPLND |  |  |
| Allaf et al. | EPLND | -0.38566 | 0.216974 |
|  | SPLND |  |  |

The Diff indicates; The Std.err indicates estimated standard error.

| Studies regarding Lymphocele rate | Sample size of each arm | | | |
| --- | --- | --- | --- | --- |
|  | LPLND  (n = 16) | EPLND  (n = 50) | SePLND  (n = 61) | SPLND  (n = 20) |
| Clark et al. | NA | NA | 123(3) | 123(1) |
| Yuh et al. | 204(6) | 202(5) | NA | NA |
| Morizane et al. | 902(6) | NA | 431(15) | NA |
| Mistretta et al. | NA | 75(7) | NA | 109(7) |
| Maderthaner et al. | NA | 485(36) | 268(27) | NA |
| Kim et al. | NA | NA | 170(4) | 294(1) |
| Arenas et al. | 381(4) | 163(2) | NA | NA |
| Heidenreich et al. | NA | NA | 103(9) | 100(9) |
| Jung et al. | NA | NA | 45(3) | 155(2) |
| Katz et al. | NA | 62(0) | 32(0) | NA |

The number indicate the total patients and the number in brackets are patients with positive outcome.

| Studies regarding Thromboembolic rate | Sample size of each arm | | | |
| --- | --- | --- | --- | --- |
|  | LPLND  (n = 8) | EPLND  (n = 8) | SePLND  (n = 11) | SPLND  (n = 6) |
| Clark et al. | NA | NA | 123(2) | 123(0) |
| Yuh et al. | 204(6) | 202(2) | NA | NA |
| Maderthaner et al. | NA | 485(5) | 268(5) | NA |
| Arenas et al. | 381(2) | 163(1) | NA | NA |
| Heidenreich et al. | NA | NA | 103(4) | 100(6) |

The number indicate the total patients and the number in brackets are patients with positive outcome.

| Studies regarding Overall complication rate | Sample size of each arm | | | |
| --- | --- | --- | --- | --- |
|  | LPLND  (n = 83) | EPLND  (n = 133) | SePLND  (n =100) | SPLND  (n = 50) |
| Touijer et al. | 700(51) | 740(47) | NA | NA |
| Yuh et al. | 204(14) | 202(9) | NA | NA |
| Morizane et al. | 902(15) | NA | 431(19) | NA |
| Mistretta et al. | NA | 75(19) | NA | 109(34) |
| Maderthaner et al. | NA | 485(33) | 268(27) | NA |
| Kim et al. | NA | NA | 170(20) | 294(7) |
| Stone et al. | 150(3) | NA | 39(14) | NA |
| Heidenreich et al. | NA | NA | 103(9) | 100(9) |
| Katz et al. | NA | 62(25) | 32(11) | NA |

The number indicate the total patients and the number in brackets are patients with positive outcome.

**The analytic code:**

install.packages("gemtc")

install.packages("rJava")

install.packages("xlsx")

library("gemtc")

library("rJava")

library("xlsx")

setwd("C:/Users/xianluzhang/Desktop/data")

getwd()

lnpos <- read.xlsx("data.xlsx", sheetName = "Lymph node positive rate", header = TRUE)

lnpos

treatments <- read.xlsx("trtdes.xlsx", sheetIndex = 1, header = TRUE)

treatments

lnpos_nw <- mtc.network(lnpos, description = "Network of postive LN", treatments = treatments)

setEPS()

postscript("LNPosnetwork.eps", width = 30, height = 15)

plot(lnpos_nw, use.description = TRUE,

vertex.label.cex = 1.5,

vertex.size = lnpos$sampleSize/30,

vertex.shapes = "circle",

vertex.label.color = "black",

vertex.label.dist = 3,

vertex.label.degree = -pi/3,

vertex.color = "darkblue",

dynamic.edge.width = TRUE,

edge.color = "gray",

vertex.label.font = 2)

dev.off()

lnpos_mdl <- mtc.model(lnpos_nw, likelihood = "binom", link = "log", type = "consistency", linearModel = "random", dic = TRUE)

lnpos_result <- mtc.run(lnpos_mdl, n.adapt = 10000, n.iter = 50000, thin = 10)

summary(lnpos_result)

dev.new()

setEPS()

postscript("LNPos轨迹图与密度图.eps", width = 30, height = 15)

plot(lnpos_result)

gelman.plot(lnpos_result)

setEPS()

postscript("LNPos森林图.eps", width = 30, height = 15)

forest(relative.effect(lnpos_result , t1 = "4"), use.description = TRUE)

lnpos_tb <- round(exp(relative.effect.table(lnpos_result)),2)

lnpos_tb

write.xlsx(lnpos_tb, "lnpos_tb.xlsx")

dev.new()

lnpos_rank <- rank.probability(lnpos_result, preferredDirection = 1)

setEPS()

postscript("LNPos概率排序图.eps", width = 30, height = 15, fonts=c("serif","Palatino"))

plot(lnpos_rank)

dev.off()

print(lnpos_rank)

dev.new()

lnpos_sucrarank <- sucra(lnpos_rank)

setEPS()

postscript("LNPos概率sucra排序图.eps", width = 30, height = 15)

plot(lnpos_sucrarank)

lnpos_ns <- summary(mtc.nodesplit(lnpos_nw, comparisons = mtc.nodesplit.comparisons(lnpos_nw),

linearModel = "random", n.adapt = 10000, n.iter = 50000, thin = 10))

dev.new()

setEPS()

postscript("LNPos一致性.eps", width = 30, height = 15)

plot(lnpos_ns)

lnpos_anohe <- summary(mtc.anohe(lnpos_nw,n.adapt = 10000, n.iter = 50000, thin = 10))

dev.new()

setEPS()

postscript("LNPos异质性分析.eps", width = 30, height = 15)

plot(lnpos_anohe)
